# A repeat length variation in *myo*-inositol monophosphatase gene contributes to seed size trait in chickpea

**DOI:** 10.1038/s41598-017-05332-x

**Published:** 2017-07-06

**Authors:** Vikas Dwivedi, Swarup Kumar Parida, Debasis Chattopadhyay

**Affiliations:** National Institute of Plant Genome Research, Aruna Asaf Ali Marg, New Delhi, 110067 India

## Abstract

Chickpea (*Cicer arietinum* L.) is the third most important food legume crop. Seed size is the most economically important trait for chickpea. To understand the genetic regulation of seed size in chickpea, the present study established a three-way association of CT repeat length variation of a simple sequence repeat (SSR) in *myo*-inositol monophosphatase gene (*CaIMP*) with seed weight and phytic acid content by large scale validation and genotyping in a set of genetically diverse germplasm accessions and two reciprocal intra-specific mapping populations. Germplasms and mapping individuals with CT repeat-length expansion in the 5′ untranslated region of *CaIMP* exhibited a pronounced increase in CaIMP protein level, enzymatic activity, seed-phytate content and seed weight. A chickpea transient expression system demonstrated this repeat-length variation influenced the translation of *CaIMP* mRNA, apparently by facilitating translation initiation. Our analyses proposed that the SSR marker derived from 5′ UTR of a *CaIMP* gene is a promising candidate for selection of seed size/weight for agronomic trait improvement of chickpea.

## Introduction

Chickpea is well known for its high protein content (24.6%) and is consumed as a substitute of animal protein^1, 2^. Despite having a narrow genetic base, chickpea shows a wide variation in seed size/weight in the germplasm collections^3^. Seed size is the most desirable trait in chickpea that determines its market price and economic importance in trade and commerce. Apart from that, chickpea seed size is also considered as an important factor for germination, growth, vigor and yield of its subsequent generation of plants^4, 5^. Therefore, seed size is considered as an important objective for improving yield and product quality in chickpea breeding programs. Seed weight was also proposed as a reliable measure of chickpea seed size^6^. Several earlier conventional genetics and breeding efforts reported various complex genetic inheritance pattern of seed size/weight such as, high^7, 8^ and low^9^ heritability for seed size, dominance of small seeds over large seeds^8, 10–12^ and vice-versa^7^. Further, seed size/weight inheritance was reported to be monogenic^13^, digenic^6, 14^, oligogenic^15^, and polygenic^7, 8, 10, 12, 16^.

In view of complex inheritance pattern and wider phenotypic variation in natural germplasm accessions, multiple genomics-assisted breeding strategies including QTL (quantitative trait loci) mapping and genetic association analysis have been deployed to identify major QTLs and candidate genes governing seed size/weight in chickpea by using intra- and inter-specific chickpea mapping populations^17–24^. A recent study reported two significant seed weight QTLs at two genomic regions, one spanning from 3.07 Mb to 4.15 Mb on LG1 and the other, from 11.12 Mb to13.82 Mb on LG4 of chickpea genome assembly^2^. In spite of the considerable progress in QTL and association mapping any potential gene regulating seed size/weight has not been delineated and utilized in marker-assisted selection (MAS) so far to develop cultivars with high seed weight and yield in chickpea.

About two-third of phosphorus in cereal and legume seeds is stored in the form of phytate [salt form of phytic acid (PA), *myo*-inositol 1,2,3,4,5,6-hexakisphosphate, IP_6_]^25^. *Myo*-inositol is a derivative of glucose. Biosynthesis of *myo*-insitol-1-phosphate from glucose-6-phosphate is catalyzed by *myo*-inositol phosphate synthase (MIPS). *Myo*-inositol monophosphatase (IMP) catalyzes the last step of *myo*-inositol biosynthesis by dephosphorylation of *myo*-insitol-1-phosphate. This enzyme is also responsible for recycling of *myo*-inositol by dephosphorylating other inositol compounds^26, 27^. Recently, repeat-length variation of an SSR (simple sequence repeat) marker (NCPGR90) in the 5′-untranslated region (5′UTR) of *CaIMP* gene (LOC101496687) was reported to exhibit 13% association with seed phytate content within a natural population of sixty germplasm accessions of chickpea^28^. A systematic effort has been made in the current study to infer a three way association of a *CaIMP* gene-derived SSR marker (NCPGR90) with seed weight (SDW) and seed-phytate content using precisely field-phenotyped germplasm accessions and reciprocal intra-specific mapping populations of chickpea. Further, using a chickpea transient system, we demonstrated that this repeat length variation in *CaIMP* influences translation efficiency of the transcript resulting in differential CaIMP protein expression.

## Results

### Repeat length variation of a SSR marker NCPGR90 contributes to chickpea seed weight

A collection of genetically diverse 71 chickpea germplasm accessions including landraces with wide geographical distribution across 20 countries of the world was gathered^20^. A wide phenotypic variation for 100-seed weight ranging from 4.53 to 70.3 g including a normal frequency distribution of target trait was observed in these accessions (Table S1 and Fig. S1A). A SSR marker (NCPGR90) with CT repeat was amplified, genotyped and sequenced among 71 accessions for assessing its potential for fragment length polymorphism across these chickpea accessions. The marker essentially detected seven different CT-repeat length variations (CT_10_, CT_11_, CT_27_, CT_29_, CT_31_, CT_32_, and CT_34_) (Fig. S1B). The SSR marker produced two major groups of amplicons with fragment size lengths of about 180 bp and about 220 bp that collectively were named as NCPGR90_180 and NCPGR90_220, respectively (Fig. S2A). Notably, seven wild accessions of *C*. *reticulatum* and *C*. *echinospermum*, belonging to chickpea primary gene pool, possessed short repeats at the SSR marker locus (Fig. S2B). More than 80% of the traditional landraces with geographical locations outside Indian subcontinent used in this study showed presence of NCPGR90_220 alleles (Table S1).

**Figure 1:**
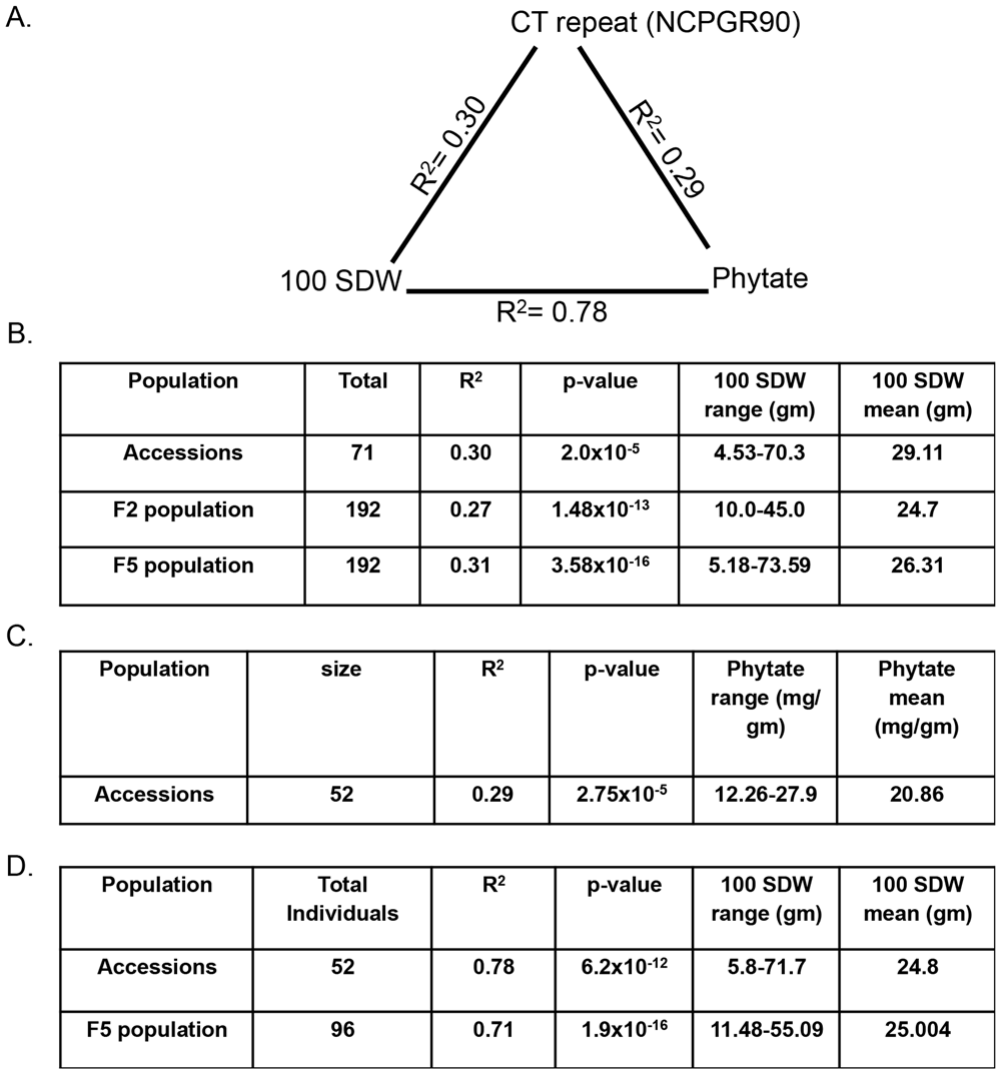
Association analysis of NCPGR90 with seed weight and phytate: Three-way association analysis of NCPGR90, 100 SDW and phytate content with each other. Association coefficients (R) are mentioned for each pair (**A**). Tables showing data of association analysis of NCPGR90 with 100 SDW in 71 chickpea germplasm accessions, F_2_ mapping population (ICCX-810800 × ICCV95334) and F_5_ mapping population (ICCV95334 × ICCX-810800) (**B**) association analysis of 100 SDW with seed phytate content (mg PA/gm dry weight) in 52 chickpea germplasm accessions and 96 individuals of F_5_ mapping population (ICCV95334 × ICCX-810800) (**C**) and association analysis of NCPGR90 with seed phytate content in 52 chickpea germplasm accessions (**D**).

The association of *CaIMP* gene-derived SSR marker (NCPGR90) with 100 SDW was studied using both GLM and MLM approaches. Using native model (GLM_marker + trait) and considering structured population (GLM_marker + trait + Q) and/or kinship by MLM, the NCPGR90 SSR marker alleles exhibited significant association with 100 SDW (R^2^ = 0.30, p = 0.00002). NCPGR90_220 and NCPGR90_180 marker alleles explained 71.9% (p = 6.1 × 10^−7^) and 28.1% (p = 2 × 10^−5^) of the total seed weight phenotypic variation observed in chickpea germplasm accessions, respectively (Fig. 1 and Table S2).

Two intra-specific reciprocal crosses were made using *kabuli* and *desi* accessions ICCV95334 (CT_31_, 100 SDW = 51.3 g) and ICCX-810800 (CT_11_, 100 SDW = 11.6 g), respectively, as parents with contrasting 100 SDW and SSR repeat-length variations. A representative set of 192 mapping individuals derived from each of F_2_ (ICCX-810800 × ICCV95334) and F_5_ (ICCV95334 × ICCX-810800) intra-specific populations exhibiting a broader 100 SDW trait variation and normal frequency distribution along with transgressive segregation of target trait (Tables S3 and S4 and Figs S3 and S4) were used to assess association of NCPGR90 SSR marker with 100 SDW following the aforesaid methods. Based on this analysis, the *CaIMP* gene-based SSR marker exhibited significant association (R^2^ = 0.27 to 0.31, p = 1.48 × 10^−13^ to 3.58 × 10^−16^) with 100 SDW in both mapping populations developed in chickpea (Fig. 1 and Table S2).

### Chickpea seed weight is strongly associated with its phytate content

Association of seed weight with seed phytate content was assessed in 52 genetically diverse chickpea germplasm accessions with 100 SDW ranging from 5.8–71.7 g and seed phytate content ranged from 12.26–27.9 mg/g seed dry weight (Table S5). A significant association (R^2^ = 0.78, p = 6.2 × 10^−12^) was observed between seed weight and its phytate content, which was further validated (R^2^ = 0.71, p = 1.9 × 10^−16^) in the F_5_ mapping population (Fig. 1 and Table S6) of (ICCV95334 × ICCX-810800). As reported before^28^, NCPGR90 showed a similar significant association (R^2^ = 0.29, p = 2.75 × 10^−5^) with seed phytate content in these 52 germplasm accessions (Fig. 1 and Table S5).

### Chickpea accessions with longer NCPGR90 repeat expressed higher CaIMP protein

**Figure 2:**
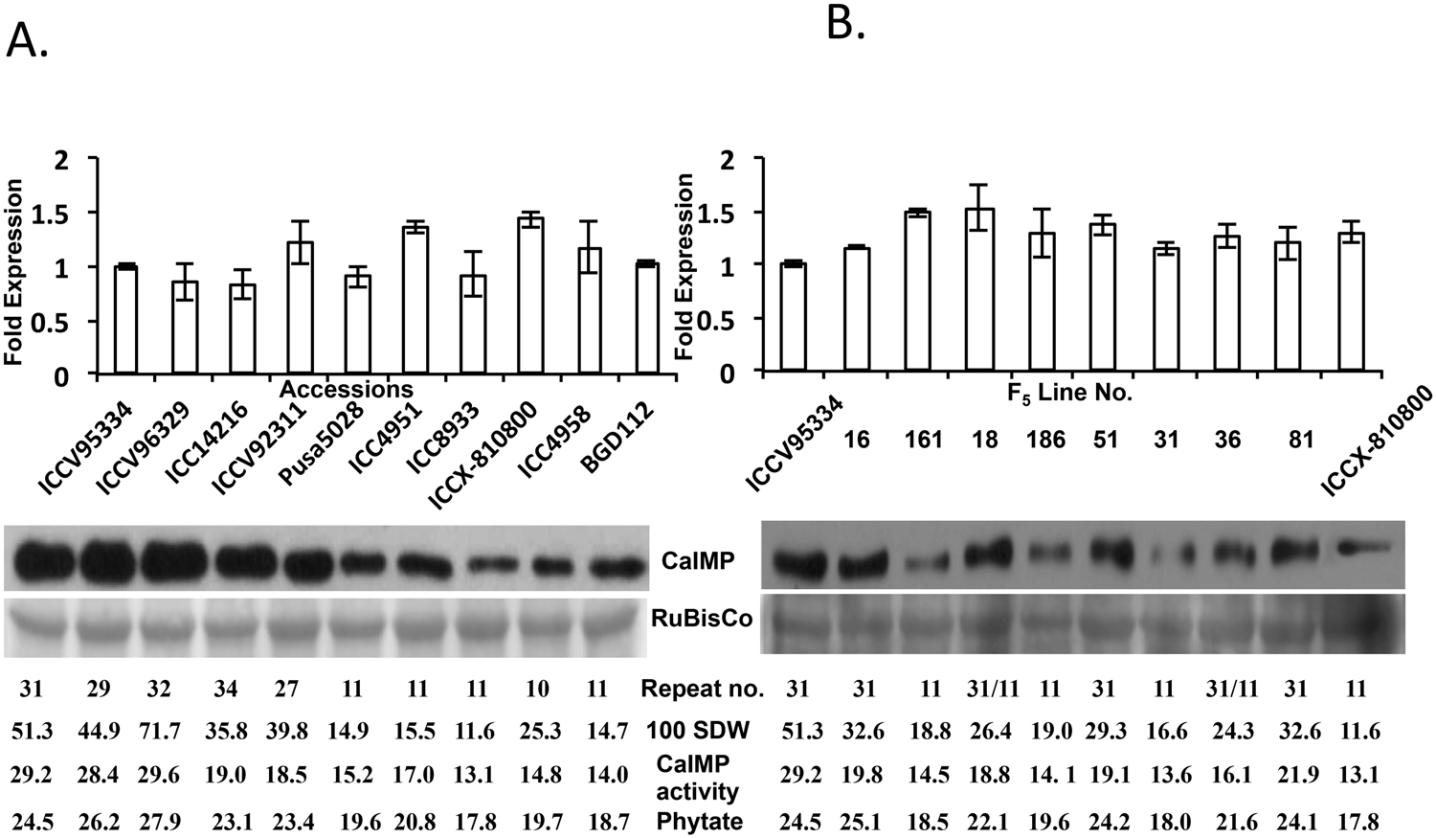
Expression analysis of CaIMP in accessions and mapping individuals: qRT-PCR analysis (top) and western blot analysis (bottom) of CaIMP gene and protein expression in 10 different chickpea germplasm accessions (**A**) and in 8 individuals of F_5_ mapping population of ICCV95334 × ICCX-810800 (**B**). CT repeat number, 100 Seed weight (100 SDW in gram), CaIMP specific activity (μmol Pi mg^−1^ protein min^−1^), and seed phytate content (mg PA/gm) of 10 chickpea germplasm accessions and 8 individuals of mapping population are shown. Chickpea Elongation factor 1-α (CaEF-1α) was used as internal control for normalization in qRT-PCR and RuBisCo was used as loading control for western blot. Standard deviation was determined from three biological replicates.

The CT repeat of NCPGR90 is located at the 5′UTR of chickpea *IMP* gene (*CaIMP*) and initiates after 68 bases from the transcription start site^29^. *CaIMP* (LOC101496687) is located between 6,150,779 bp and 6,155,608 bp on LG 1 of chickpea genome assembly and is close to one significant seed weight QTL mentioned above^30^. A representative of 10 accessions with various NCPGR90 SSR repeat length and different seed weight were randomly picked for qRT-PCR to compare transcript level of *CaIMP* gene at normal growth condition. A maximum of 1.6 fold difference in the steady-state transcript abundance of *CaIMP* gene was observed in these accessions. Similarly, eight random mapping individuals and two parents of F_5_ intra-specific population of (ICCV95334 × ICCX-810800) with homozygous and heterozygous NCPGR90 alleles and different seed weights did not show any significant variation in transcript abundance of *CaIMP* gene (Fig. 2). Several lines of evidences documented the importance of sequence and structure of 5′UTR in determining level of protein expression^31–33^. Therefore, CaIMP protein level in the same chickpea germplasm accessions and mapping individuals/parents was assessed by western blotting. Remarkably huge variation in the CaIMP protein expression level was observed in these accessions irrespective of their corresponding transcript levels, with higher CaIMP protein levels were observed in the accessions with longer CT repeats and higher seed weight. Same trend of differential CaIMP protein level was observed in the F_5_ mapping individuals and parents with different seed weight. Specific activity of CaIMP enzyme was determined in the same germplasm accessions and mapping individuals. CaIMP possesses broad substrate specificity however; it showed maximum activity with *myo*-inositol 1-phosphate in *in vitro* system^29^. *Myo*-inositol 1-phosphate was used as a substrate to measure activity of CaIMP in the germplasm accessions and mapping individuals mentioned above. CaIMP activity in these accessions/individuals ranged from 29.6–13.9 μmol of inorganic phosphate released/mg/min. Although, the fold of catalytic activity of CaIMP did not accurately follow the expression levels of CaIMP protein in these accessions/individuals, however, those expressing higher CaIMP protein exhibited higher CaIMP enzymatic activities than those having lower CaIMP protein level. CaIMP catalyzes the last step of biosynthesis of *myo*-inositol, which is a precursor of phytic acid. Therefore, phytic acid content was measured in the same accessions and F_5_ mapping individuals. Phytic acid content ranged from 27.9–17.8 mg/g of seed dry weight in these accessions/individuals. The accessions/individuals with higher CaIMP protein level and activity exhibited higher seed phytic acid content (Fig. 2).

### Repeat length variation in CaIMP gene influenced its protein level

**Figure 3:**
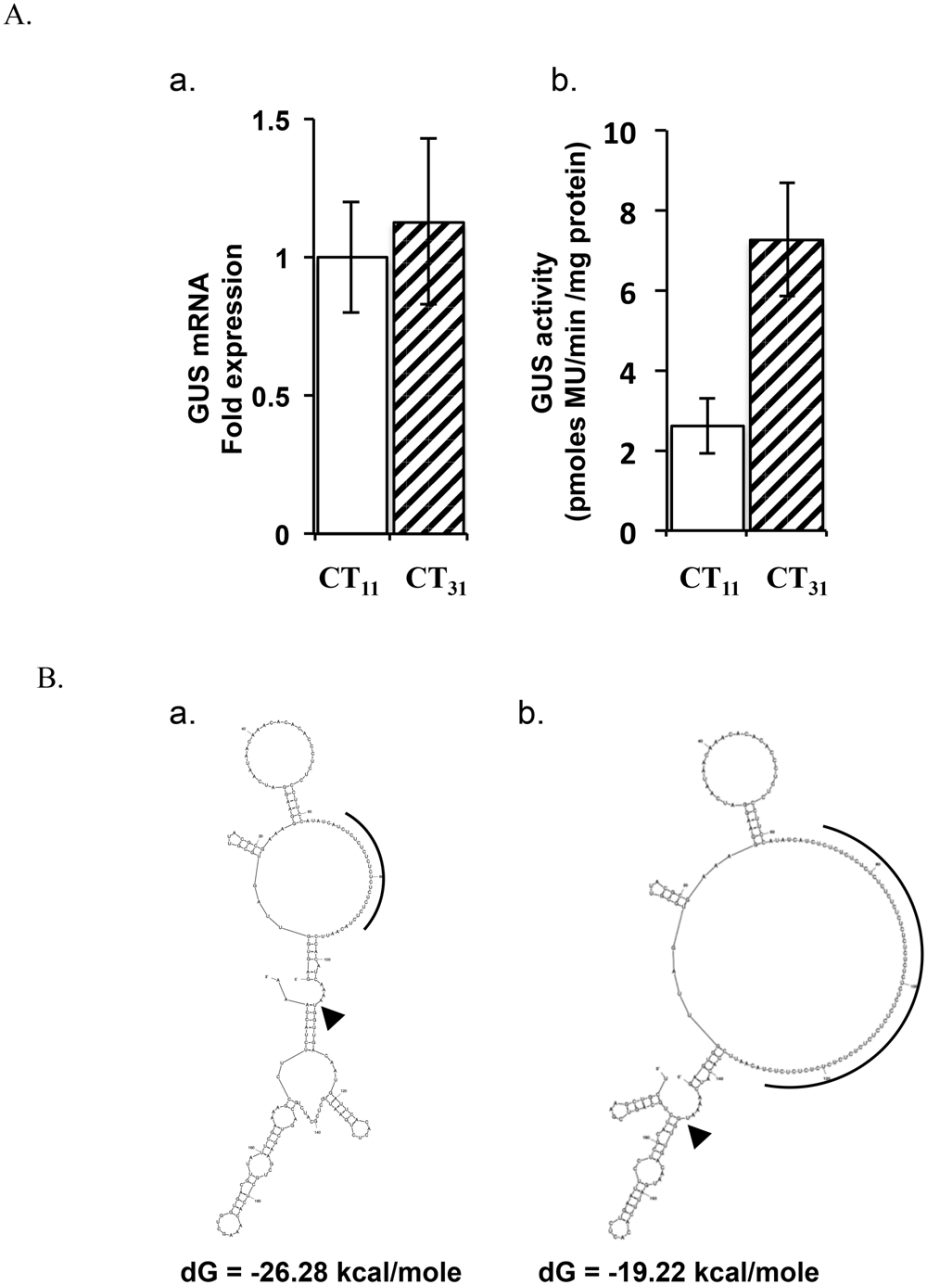
Transient expression analysis of CaIMP: (**A**) Beta-glucuronidase (GUS) was transiently expressed by agroinfiltration in young leaves of chickpea ICCV95334 under the control of 1.5 kb *CaIMP* promoter and 5′UTR containing CT_11_ or CT_31_. GUS mRNA level was assessed by qRT-PCR. CaEF-1α gene was used as internal control (a). Specific activity of GUS enzyme was determined for GUS protein expression (b). Standard deviation was determined from three biological replicates. Fold expression of a Green fluorescence protein (GFP) gene from a plasmid agroinfiltrated in two leaf samples simultaneously with the GUS constructs was determined by qRT-PCR and was used to normalize transformation efficiency. (**B**) Secondary structure and folding energy (dG) of first 200 base *CaIMP* mRNA from (a) ICCX-810800 (CT_11_) (b) ICCV95334 (CT_31_) was predicted using mfold tool. The CT repeat and the translation start site are shown by arc and arrow, respectively.

Variation in CaIMP protein level in different chickpea germplasms could have also resulted from the variation in the *CaIMP* transcript level and/or due to their different genetic background. Therefore, a transient expression system was used to investigate any role of length variation in CT repeat within the 5′UTR of *CaIMP* gene in its expression under the same genetic background. A 1.5 kb DNA fragment upstream to the translation start site of *CaIMP* having CT_31_ or CT_11_ was inserted in to a binary plasmid to drive expression of GUS reporter gene. These constructs were used to transiently transform young chickpea leaves of one mapping parent ICCV95334 along with another plasmid expressing GFP reporter gene for normalization of transformation efficiency. Normalized transcript abundance of GUS gene from two constructs varying only in CT repeat length did not show any significant difference. However, normalized GUS activity, a measure of GUS protein expression level, in the samples transformed with the construct having CT_31_ was about 2.7-fold higher than that in the samples transformed with the construct having CT_11_ in three biological replicates (Fig. 3A) suggesting that the CT repeat length variation in *CaIMP* 5′UTR affected translation instead of transcription.

Folding of mRNA at the beginning of genes is one of the major factors that affect translation initiation and, thereby, protein level. Lower structural stability (higher folding energy) of mRNA near the translation start codon was suggested to favor translation initiation^34–36^. The secondary structure and folding energy of *CaIMP* mRNA was predicted using mfold tool^37^. Predicted folding energy (dG) of the first 200 nt of *CaIMP* mRNA with CT_31_was much higher (−19.22 kcal/mol) than that of the mRNA with CT_11_ (−26.28 kcal/mol) (Fig. 3B) suggesting that CaIMP mRNA with CT_31_ would be more favored for translation initiation than that with CT_11_.

## Discussion

In the present study, we used a set of diverse chickpea germplasm accessions and mapping populations derived from two intra-specific reciprocal crosses of two accessions with contrasting seed weight trait to propose that length variation of an SSR in the 5′UTR of *CaIMP* gene is strongly associated with seed size/weight. Previously, the same repeat element was shown to be moderately associated with phytate content and drought tolerance using a limited set of germplasm accessions of chickpea. In the previous report, repeat length variation of the SSR marker was correlated with differential transcript level of *CaIMP* in a genetically diverse set of chickpea accessions. However, transcript level of a gene in different accessions may vary due to various reasons including differential activities of the proteins regulating transcription of that gene and different genetic backgrounds of the accessions. In this study, we have demonstrated using a transient chickpea system in a single genetic background that the SSR repeat length variation affected rather translation than transcription of *CaIMP*. We have also shown that CaIMP protein level and its enzymatic activity corresponded well with the CT-repeat length, seed phytate level and seed weight in natural population of chickpea.

A SNP-based genome-wide genotyping approach to identify major QTLs governing chickpea seed weight has delineated a genomic region spanning from 3.07 Mb to 4.15 Mb on LG1. However, none of the five SNPs identified at this QTL region was in predicted genes probably due to narrow candidate region^2^. The *CaIMP* gene that resides within 2 Mb of this region (LOC101496687, CaLG01: 6,150,779–6,155,608) could be a potential candidate for this QTL responsible for seed weight variation in chickpea.

Phytic acid is a ubiquitous storage form of phosphate in eukaryotes. It regulates many physiological functions such as stress response, development, phosphate sensing and homeostasis^38^. Phosphates released from phytates are important for signal transduction, DNA synthesis and regeneration of adenosine triphosphate (ATP) and thus making it an essential energy source for seeds^39^. Previously, in cereals, mutations in various other genes were shown to reduce phytate content and seed weight^40–42^. Here we propose that one of the mechanisms that natural chickpea population use to regulate seed size and phytate content is through variation in CT repeat length at the 5′UTR of the *CaIMP* gene and thus, by variation in CaIMP protein level. Although adequate dietary phosphate is essential for health, a reduction in phytic acid content in cereal and legume grains is desired because besides sequestering inorganic phosphate, phytic acid chelates essential minerals causing health problem^43, 44^. Several attempts were made before to reduce phytic acid content in seeds by isolating low phytic acid mutants and raising transgenic plants. However, these efforts were usually accompanied with compromised agronomic performance of the seeds and plants^45, 46^. Varietal difference of IMP protein expression due to repeat length variation in the 5′UTR and associated seed size variation have not been reported in other plants. 5′UTR of the corresponding *IMP* gene (Medtr2g026060) in *Medicago truncatula* possesses CT_5_ whereas, its orthologs in soybean (Glyma15g115500) and common bean (Phvul03g084500) showed presence of CT_2_CA_3_ and CTCACT_2_, respectively, in the similar positions^47–49^. Therefore, it appears that presence of CT/CA repeats in the 5′UTR of *IMP* gene is an evolutionary feature in legumes, particularly, in chickpea.

To summarize, we have shown a three-way association between a polymorphic SSR at the 5′UTR of *CaIMP* gene, seed weight and seed phytate content in chickpea using a set of genetically diverse germplasm accessions and two mapping populations derived from reciprocal crosses. Chickpea germplasm accessions and mapping individual with longer CT repeat exhibited higher CaIMP protein level and enzymatic activity, and seed weight and phytate content. Using chickpea transient system we have shown that CT repeat length variation influenced translation of *CaIMP* gene, and mRNA fold energy predicted a preferred translation initiation for *CaIMP* mRNA with longer CT repeat. Phytate content is reported to regulate many physiological properties including seed viability, and increased seed phytate level has negative effects on health. High chickpea seed weight is a desirable market trait. Therefore, CT repeat length variation at the 5′UTR of *CaIMP* can be used as a valuable marker for selection of chickpea lines with desirable seed trait without compromising the plant performance.

## Methods

Seventy one germplasm accessions representing various chickpea agro-ecological growing areas of the world, collected from International Crop Research Institute for Semi-Arid Tropics (ICRISAT), India were used for genetic association analysis. Additionally, 192 mapping individuals represented from each of a F_2_ and a F_5_ intra-specific mapping population (*desi* accession ICCX-810800 × *kabuli* accession ICCV95334) and (ICCV95334 × ICCX-810800), respectively with contrasting 100-seed weight trait were included. These germplasm accessions and mapping individuals along with mapping parental accessions were phenotyped for 100-seed weight (g) in field at two geographical locations (ICRISAT and Delhi) in India as described before^20^. DNA was extracted from fresh leaf tissues of one month-old plants grown in the field using the cetyltrimethyl ammonium bromide (CTAB) method^50^. All the germplasm accessions and mapping individuals were genotyped with the SSR marker (NCPGR90) using the primer pair Ca14825.1 F: 5′-TATAGAGAGAGAAAGAGAGAGG-3′; Ca14825.1R: 5′-CTAAGAGCACATACGGTTTTGT-3′. Quantitative real-time PCR (qRT-PCR) was carried out according to the procedure described before^51^. The expression of CaIMP mRNA was calculated according to delta-delta Ct method of the system. Chickpea Elongation factor 1-α (EF-1α) was used as internal control for normalization. All the primers used are mentioned in Text S1. The protein extraction, partial purification and IMP assay was performed as described before^29^. IMP enzyme activity assay was performed by colorimetric estimation of released inorganic phosphate (Pi)^52^. Polyclonal antibody corresponding to IMP protein was raised in rabbit using bacterially expressed protein. Rabbit polyclonal antibody against IMP (1:1,000 dilution) was used as primary antibody and the western blot was probed with horseradish peroxidase-conjugated donkey anti-rabbit-IgG secondary antibody (1:10,000) (Amersham, Little Chalfont, UK). Bands were detected using ECL reagent (Promega, Madison, USA) following manufacturer’s instructions. Phytic acid (PA) content was estimated according to previously described method^53^. To determine the association potential of *IMP* gene-derived SSR marker with seed weight trait, the marker genotyping data across accessions were subjected to the non-parametric Kruskal-Wallis one-way ANOVA using PAST version 3.0 software^54^. The detailed association analysis was performed using the marker genotyping and 100-seed weight field phenotyping data of 71 chickpea germplasm accessions and 192 representative mapping individuals from each of F_2_ and F_5_ intra-specific populations. An association test between SSR marker and 100-seed weight (g) was performed using a general linear model (GLM) and subsequently by a mixed linear model (MLM) that relied on both population structure (Q) as well as kinship (K) statistics^55^. The degree of association of *IMP* gene-based SSR marker with seed-weight trait was estimated by the R^2^ using a model with the SSR and adjusted P-value following a false discovery rate (FDR-controlling method. The SSR marker exhibiting significant association with seed weight trait at the lower FDR adjusted P-value (threshold P < 10^−5^) and higher R^2^ (degree of SSR marker-trait association) was considered for further analysis in chickpea.

For transient gene expression analysis, the 1.5 kb upstream sequences of *IMP* gene upto the translation start codon were amplified from two low and high seed weight chickpea mapping parental accessions ICCV95334 and ICCX-810800 and cloned in pCAMBIA1301 vector to direct expression of beta-glucuronidase (GUS) reporter protein. The GUS gene in this plasmid possesses an intron for detecting GUS expression from plant cells only. *Agrobacterium tumefaciens* strain EHA105 cultures (OD_600_ 0.6–0.8) carrying this recombinant plasmid and pCAMBIA1302 plasmid having GFP were mixed equally, resuspended in infiltration medium (10 mM Mgcl_2_, 10 mM MES, 200 µM acetosyringone) and infiltrated in to young leaves of two-month-old chickpea ICCV95334. The leaves were kept in dark for twelve hours after infiltration and expression of the reporter genes was assessed after three days. GUS activity was estimated by monitoring cleavage of the substrate 4-methylumbelliferyl β-D-glucuronide (MUG). Transformation efficiency was normalized by fold change in GFP transcript expression measured by qRT-PCR.

## Electronic supplementary material

Supplementary Information
