# Supplementary material for: A repeat length variation in myo-inositol monophosphatase gene contributes to seed size trait in chickpea
Source: Sci Rep. 2017 Jul 6;7:4764. doi: 10.1038/s41598-017-05332-x (PMC5500587; doi:10.1038/s41598-017-05332-x)
Supplement: Supplementary file 1 — Supplementary Information [file 41598_2017_5332_MOESM1_ESM.pdf]

A repeat length variation in *myo*-inositol monophosphatase gene contributes to seed size trait in chickpea

Vikas Dwivedi, Swarup Kumar Parida* and Debasis Chattopadhyay*

Supplementary Information

A.

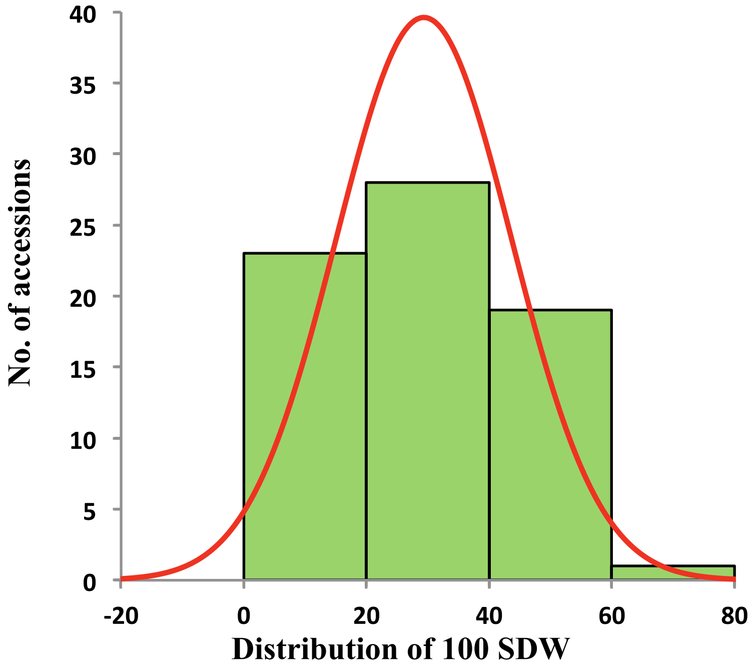

B.

```
    ICC4958   GTTAGTGCGTTACGCGAAAGGAAGGATCAATAACAAACACACACCCTCTCCCT
ICCX-810800   GTTAGTGCGTTACGCGAAAGGAAGGATCAATAACAAACACACACCCTCTCCCT
     BG5028   GTTAGTGCGTTACGCGAAAGGAAGGATCAATAACAAACACACAGCCTCTTCCT
  ICCV96329   GTTAGTGCGTTACGCGAAAGGAAGGATCAATAACAAACACACAGCCTCTTCCT
  ICCV95334   GTTAGTGCGTTACGCGAAAGGAAGGATCAATAACAAACACACAGCCTCTTCCT
   ICC14216   GTTAGTGCGTTACGCGAAAGGAAGGATCAATAACAAACACACAGCCTCTTCCT
   ICC92311   GTTAGTGCGTTACGCGAAAGGAAGGATCAATAACAAACACACAGCCTCTTCCT

    ICC4958   TCCATATCATCTCTCTCTCTCTCTCTCTCT.......................
ICCX-810800   TCCATATCATCTCTCTCTCTCTCTCTCTCTCT.....................
     BG5028   TCCATATCATCTCTCTCTCTCTCTCTCTCTCTCTCTCTCTCTCTCTCTCTCTC
  ICCV96329   TCCATATCATCTCTCTCTCTCTCTCTCTCTCTCTCTCTCTCTCTCTCTCTCTC
  ICCV95334   TCCATATCATCTCTCTCTCTCTCTCTCTCTCTCTCTCTCTCTCTCTCTCTCTC
   ICC14216   TCCATATCATCTCTCTCTCTCTCTCTCTCTCTCTCTCTCTCTCTCTCTCTCTC
   ICC92311   TCCATATCATCTCTCTCTCTCTCTCTCTCTCTCTCTCTCTCTCTCTCTCTCTC


    ICC4958   .........................ACAATTCCACATCAAAATGGTTGA
ICCX-810800   .........................ACAATTCCACATCAAAATGGTTGA
     BG5028   TCTCTCTCTCT..............ACAATTCCACATCAAAATGGTTGA
  ICCV96329   TCTCTCTCTCTCTCT..........ACAATTCCACATCAAAATGGTTGA
  ICCV95334   TCTCTCTCTCTCTCTCTCT......ACAATTCCACATCAAAATGGTTGA
   ICC14216   TCTCTCTCTCTCTCTCTCTCT....ACAATTCCACATCAAAATGGTTGA
   ICC92311   TCTCTCTCTCTCTCTCTCTCTCTCTACAATTCCACATCAAAATGGTTGA
```

Figure S1: A. Frequency distribution of 100 SDW (seed weight) and **B.** Representative profile of length variation of SSR NCPGR90 in 71 chickpea germplasm accessions.

A.

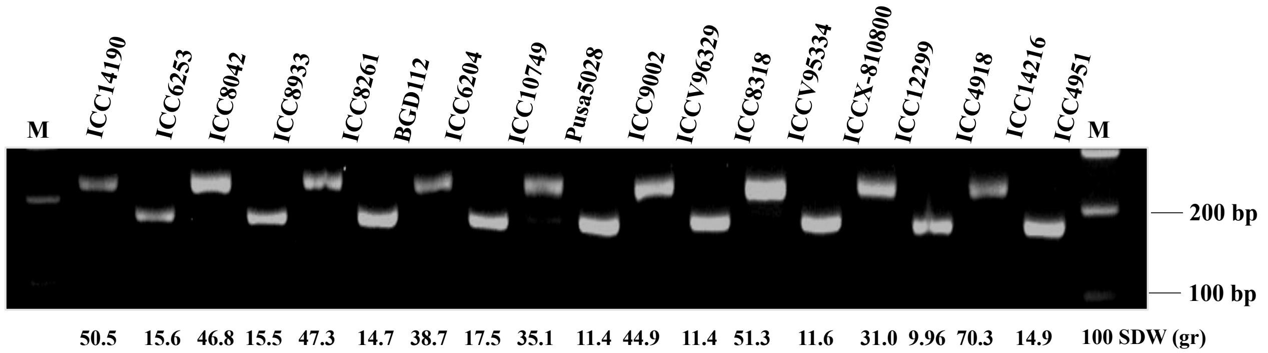

B.

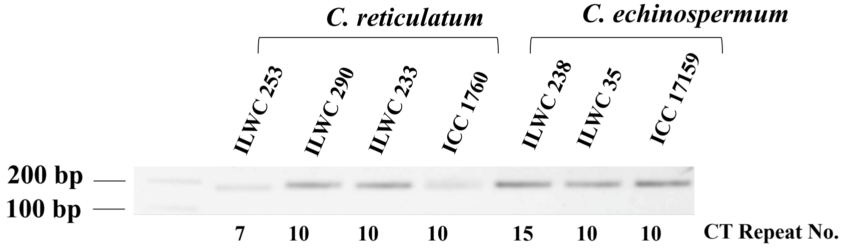

Figure S2: A. Representative profile of agarose gel electrophoresis for length variation of SSR NCPGR90 in chickpea germplasm accessions. Corresponding 100 SDWs (gm) are mentioned at the bottom. **B.** Representative profile of agarose gel electrophoresis for length variation of SSR NCPGR90 in seven wild chickpea germplasm accessions. The species names and CT repeat number are mentioned in top and bottom, respectively. 100 bp ladder is used as standard.

A.

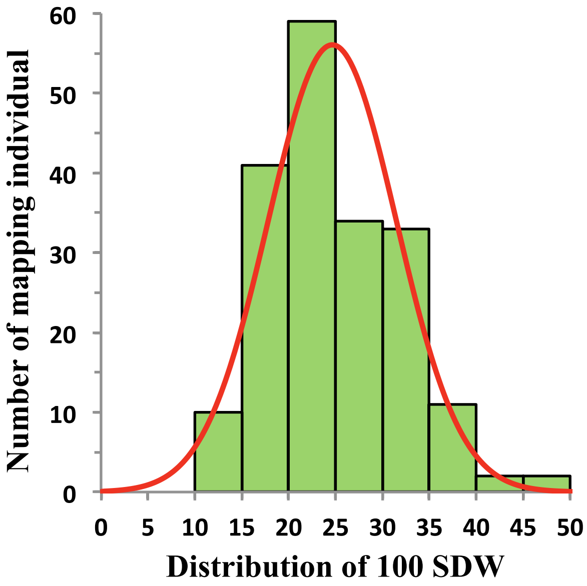

B.

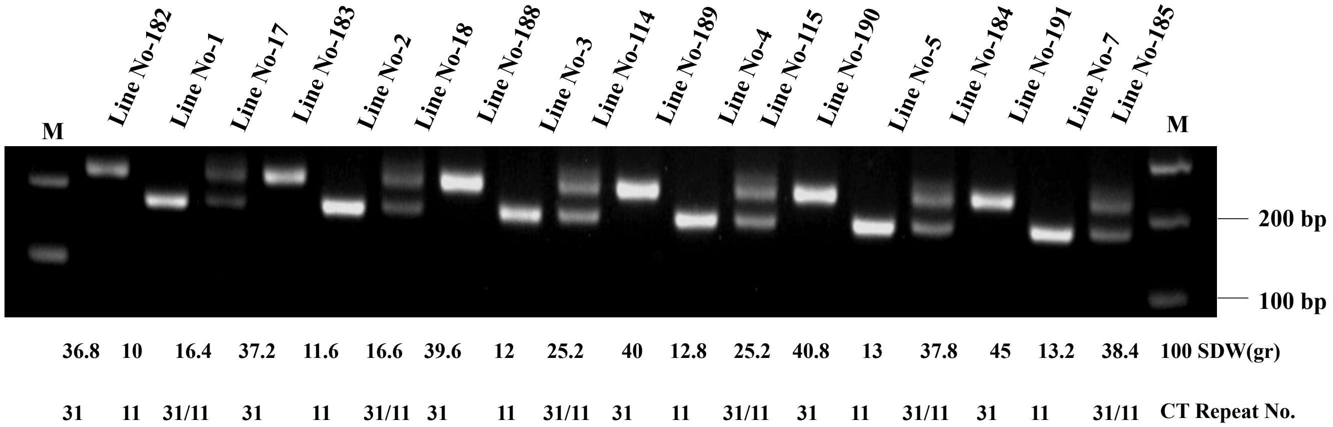

Figure S3: A. Frequency distribution of 100 SDW (seed weight) and **B.** Representative profile of agarose gel electrophoresis for length variation of SSR NCPGR90 in F2 mapping population of ICCX-810800 × ICCV95334. Corresponding 100 SDW (g) and CT repeat numbers of individual line are mentioned. 100 bp ladder is used as standard.

A.

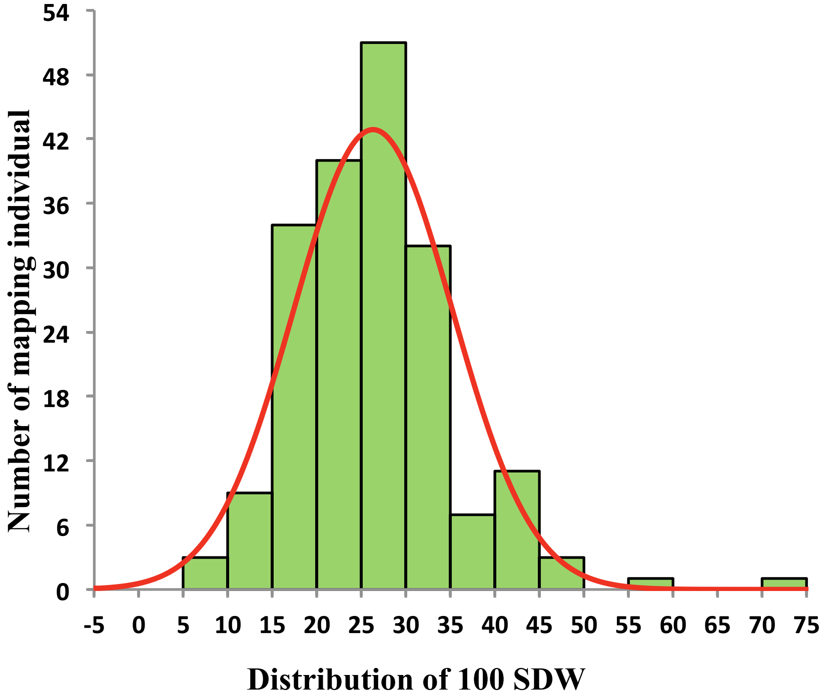

B.

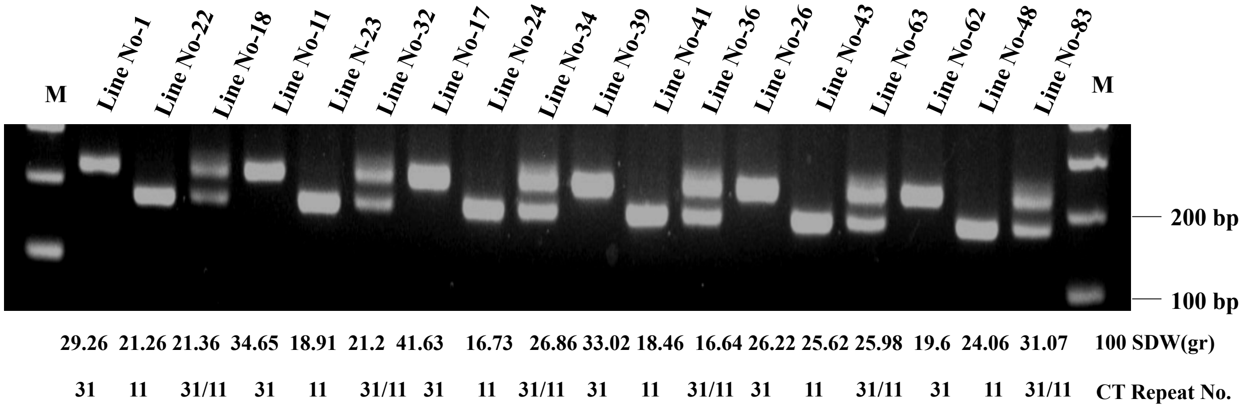

Figure S4: A. Frequency distribution of 100 SDW (seed weight) and **B.** Representative profile of agarose gel electrophoresis for length variation of SSR NCPGR90 in F5 mapping population of ICCV95334 × ICCX-810800 . Corresponding 100SDW (g) and CT repeat numbers of mapping individuals are mentioned. 100 bp ladder is used as standard.

Table S1: Seventy-one chickpea germplasm accessions used for association study.

Accession Names	Cultivar types	Geographical origin	Biological status	100 SDW (gm)	NCPGR90 allele
**BGD112**	*Desi*	Northern India	Genetic stock	14.7	180
**BGD72**	*Desi*	Northern India	Genetic stock	48.12	220
**G130**	*Desi*	India	Released Cultivar	36.67	220
**IC296131**	*Desi*	Northern India	Variety	8.97	180
**IC296132**	*Desi*	Northern India	Variety	4.53	180
**IC296133**	*Desi*	Northern India	Variety	27	220
**ICC10749**	*Kabuli*	Turkey	Traditional cultivar/Landrace	17.5	180
**ICC10755**	*Kabuli*	Turkey	Traditional cultivar/Landrace	40.91	220
**ICC10884**	*Kabuli*	Ethiopia	Traditional cultivar/Landrace	37.6	220
**ICC11301**	*Kabuli*	United States of America	Traditional cultivar/Landrace	50	220
**ICC11498**	*Desi*	India	Traditional cultivar/Landrace	17.66	220
**ICC11742**	*Kabuli*	Chile	Traditional cultivar/Landrace	31.2	180
**ICC11749**	*Kabuli*	Chile	Traditional cultivar/Landrace	20.62	220
**ICC11847**	*Kabuli*	Chile	Traditional cultivar/Landrace	29.66	220
**ICC11944**	*Desi*	Nepal	Traditional cultivar/Landrace	19.19	220
**ICC12028**	*Desi*	Mexico	Traditional cultivar/Landrace	19.68	220
**ICC12299**	*Desi*	Nepal	Traditional cultivar/Landrace	31.03	220
**ICC12328**	*Kabuli*	Cyprus	Traditional cultivar/Landrace	43.73	220
**ICC12968**	*Kabuli*	Southern India	Variety	28.5	220
**ICC13523**	*Kabuli*	Iran	Traditional cultivar/Landrace	35.78	220
**ICC14190**	*Kabuli*	India	Traditional cultivar/Landrace	50.49	220
**ICC14199**	*Kabuli*	Mexico	Breeding material	22	220
**ICC14203**	*Kabuli*	Mexico	Traditional cultivar/Landrace	29.6	220
**ICC14216**	*Kabuli*	Mexico	Breeding/Research material	71.71	220
**ICC14446**	*Kabuli*	Italy	Traditional cultivar/Landrace	40.71	220
**ICC15264**	*Kabuli*	Iran	Traditional cultivar/Landrace	34.79	220
**ICC15333**	*Kabuli*	Iran	Traditional cultivar/Landrace	32.96	220
**ICC15435**	*Kabuli*	Morocco	Traditional cultivar/Landrace	40.62	220
**ICC15512**	*Kabuli*	Morocco	Traditional cultivar/Landrace	42.74	220
**ICC15518**	*Kabuli*	Morocco	Traditional cultivar/Landrace	20.72	220
**ICC15551**	*Kabuli*	Australia	Traditional cultivar/Landrace	44.37	220
**ICC15610**	*Desi*	India	Traditional cultivar/Landrace	9.41	220
**ICC15725**	*Kabuli*	Syrian Arab Republic	Traditional cultivar/Landrace	43.63	180
**ICC15802**	*Kabuli*	Syria	Traditional cultivar/Landrace	32.72	220
**ICC16374**	*Desi*	Malawi	Breeding material	16.33	220

**ICC16811**	*Kabuli*	Portugal	Traditional cultivar/Landrace	21.31	220
**ICC16814**	*Kabuli*	Portugal	Traditional cultivar/Landrace	22.74	220
**ICC2072**	*Desi*	India	Traditional cultivar/Landrace	27.14	220
**ICC4657**	*Desi*	India	Traditional cultivar/Landrace	33.85	180
**ICC4918**	*Desi*	India	Advanced cultivar	9.96	180
**ICC4926**	*Desi*	India	Breeding/Research material	46.1	220
**ICC4951**	*Desi*	Central India	Landrace	14.9	180
**ICC4958**	*Desi*	Central India	Traditional cultivar/Landrace/genetic stock	25.3	180
**ICC5002**	*Desi*	India	Breeding/Research material	7.44	180
**ICC5590**	*Desi*	India	Breeding/Research material	15.09	220
**ICC6013**	*Desi*	India	Breeding/Research material	9.91	180
**ICC6204**	*Kabuli*	Spain	Traditional cultivar/Landrace	38.74	220
**ICC6210**	*Kabuli*	Spain	Traditional cultivar/Landrace	32.67	220
**ICC6253**	*Kabuli*	Morocco	Traditional cultivar/Landrace	15.6	180
**ICC7184**	*Desi*	Turkey	Traditional cultivar/Landrace	12.18	220
**ICC7295**	*Kabuli*	Tunisia	Traditional cultivar/Landrace	42	220
**ICC7308**	*Kabuli*	Peru	Traditional cultivar/Landrace	36.32	220
**ICC7654**	*Kabuli*	Turkey	Traditional cultivar/Landrace	27.88	220
**ICC8042**	*Kabuli*	Iran	Traditional cultivar/Landrace	46.84	220
**ICC8261**	*Kabuli*	Turkey	Traditional cultivar/Landrace	47.28	220
**ICC8318**	*Desi*	India	Traditional cultivar/Landrace	11.39	180
**ICC8933**	*Desi*	Northern India	Genetic stock	15.53	180
**ICC9002**	*Desi*	Iran	Traditional cultivar/Landrace	11.39	180
**ICC9737**	*Desi*	Afghanistan	Traditional cultivar/Landrace	9.61	220
**ICCC37**	*Desi*	Southern India	Variety	38.74	220
**ICCV1**	*Kabuli*	Southern India	variety	15.6	180
**ICCV10**	*Desi*	India	Released Cultivar	5.17	180
**ICCV92311**	*Kabuli*	India	Released Cultivar	35.80	220
**ICCV92944**	*Desi*	Central India	Variety	30.89	220
**ICCV93954**	*Desi*	Southern India	Variety	52.51	220
**ICCV95334**	*Kabuli*	Central India	Variety	51.29	220
**ICCV96329**	*Kabuli*	Southern India	Variety	44.90	220
**ICCX-810800**	*Desi*	Northern India	Variety	11.62	180
**Pusa5023**	*Kabuli*	Northern India	Variety	32.01	220
**Pusa5028**	*Desi*	Northern India	Variety	39.80	220
**Pusa547**	*Desi*	Northern India	Variety	40.57	220

Table S2: Association of SSR NCPGR90 alleles with 100 Seed Weight based on Kruskal-Wallis one-way ANOVA

SSR marker allele size bp	Germplasm accessions (71)			Mapping population F2 (192) (ICCX-810800 X ICCV95334)			Mapping population F5 (192) (ICCV95334 X ICCX-810800)		
	Hc-value	F-value	p	Hc-value	F-value	p	Hc-value	F-value	p
180	27.77	31.73	1.7×10^{-8}	86.26	98.59	3.1×10^{-23}	62.26	71.25	3.3×10^{-17}
220	77.26	88.29	5.6×10^{-21}	60.76	69.44	7.8×10^{-17}	143.3	163.7	1.7×10^{-37}
180/220				138.8	158.6	2.3×10^{-36}	80.26	91.72	9.9×10^{-22}

Table S3: 100 SDW and NCPGR90 alleles of 192 individuals of F2 (ICCX-810800 × ICCV95334) mapping population used for association study.

Line No	100 SDW (gm)	NCPGR90 allele	Line No	100 SDW (gm)	NCPGR90 allele
1	10.0	180	97	24.0	180
2	11.6	180	98	24.0	220
3	12.0	180	99	24.0	220
4	12.8	180	100	24.0	180/220
5	13.0	180	101	24.0	180/220
6	13.0	180/220	102	24.2	180/220
7	13.2	180	103	24.3	180
8	13.6	180	104	24.4	180
9	14.0	180/220	105	24.4	180/220
10	14.8	180	106	24.5	180/220
11	15.0	180	107	24.6	220
12	15.4	180	108	24.8	180
13	16.0	180	109	24.8	220
14	16.0	180	110	24.8	220
15	16.2	180	111	25.0	180/220
16	16.4	180	112	25.2	180
17	16.4	180/220	113	25.2	220
18	16.6	180/220	114	25.2	180/220
19	16.8	180	115	25.2	180/220
20	16.8	180	116	25.2	180/220
21	17.0	180	117	25.2	180/220
22	17.0	180/220	118	25.3	180
23	17.0	180/220	119	25.3	180/220
24	17.0	180/220	120	25.4	180/220
25	17.2	180/220	121	25.6	220
26	17.5	180	122	25.8	180/220
27	17.5	180/220	123	26.0	180
28	17.6	180/220	124	26.0	180/220
29	17.6	180/220	125	26.0	180/220
30	17.8	180	126	26.2	180/220
31	17.8	180	127	26.2	180/220
32	18.0	180/220	128	26.4	180/220
33	18.2	180	129	26.4	180/220
34	18.4	180	130	26.8	180/220

35	18.4	180	131	27.0	220
36	18.4	180	132	27.2	220
37	18.5	180	133	27.6	180/220
38	18.6	180	134	28.0	180/220
39	18.6	180	135	28.2	220
40	18.8	180/220	136	28.4	220
41	19.0	180	137	28.4	180/220
42	19.0	180	138	28.8	220
43	19.0	180/220	139	28.8	180/220
44	19.0	180/220	140	28.8	180/220
45	19.2	180	141	28.9	220
46	19.2	180	142	29.6	220
47	19.4	180	143	29.6	180/220
48	19.4	180/220	144	29.8	180/220
49	19.6	180	145	30.0	180
50	19.6	180/220	146	30.0	220
51	19.6	180/220	147	30.0	220
52	20.0	180	148	30.2	220
53	20.0	180	149	30.4	220
54	20.0	180/220	150	30.4	180/220
55	20.0	180/220	151	30.4	180/220
56	20.0	180/220	152	30.6	180
57	20.0	180/220	153	30.8	220
58	20.3	180/220	154	30.8	220
59	20.4	180/220	155	31.0	180/220
60	20.8	180	156	31.0	180/220
61	20.8	220	157	31.2	220
62	20.8	180/220	158	31.2	220
63	21.0	220	159	31.2	180/220
64	21.2	180	160	31.5	220
65	21.4	180/220	161	31.6	180
66	21.5	220	162	31.6	220
67	21.6	180	163	31.6	220
68	21.6	180/220	164	31.8	180/220
69	21.8	180/220	165	32.0	180/220
70	21.9	180/220	166	32.0	180/220
71	22.0	180	167	32.4	220
72	22.0	180	168	32.4	180/220
73	22.0	180	169	32.8	220

74	22.0	180	170	33.0	180/220
75	22.0	180	171	33.2	220
76	22.0	180/220	172	34.0	180/220
77	22.0	180/220	173	34.0	180/220
78	22.3	220	174	34.0	180/220
79	22.4	180/220	175	34.0	180/220
80	22.5	180/220	176	34.2	220
81	22.6	180/220	177	34.8	180/220
82	22.6	180/220	178	35.2	180
83	23.0	180	179	35.2	180/220
84	23.0	180/220	180	36.0	180/220
85	23.0	180/220	181	36.5	180/220
86	23.2	180	182	36.8	220
87	23.2	180/220	183	37.2	220
88	23.4	220	184	37.8	180/220
89	23.4	180/220	185	38.4	180/220
90	23.5	180	186	39.0	180/220
91	23.6	180	187	39.2	180/220
92	23.6	180/220	188	39.6	220
93	23.6	180/220	189	40.0	220
94	23.7	180/220	190	40.8	220
95	23.8	220	191	45.0	220
96	23.8	180/220	192	45.0	180/220

Table S4: 100 SDW and NCPGR90 alleles of 192 individuals of F5 mapping population of (ICCV95334 X ICCX-810800) used for association study.

Line No.	100 SDW (gm)	NCPGR90 allele	Line No.	100 SDW (gm)	NCPGR90 allele
1	29.26	220	237	14.6	180
2	31.44	220	238	28.14	180/220
6	30.35	220	239	26.07	220
7	26.89	220	251	23.4	180
8	18.48	220	253	33.29	180/220
10	18.26	180	254	29.35	180/220
11	34.65	220	256	22.86	220
16	32.59	220	259	30.07	220
17	41.63	220	260	27.71	220
18	26.37	180/220	266	16.68	180/220
21	23.04	220	267	19.72	180/220
22	21.26	180	270	28.93	180/220
23	18.91	180	284	38.91	220
24	16.73	180	285	73.59	220
26	26.22	220	296	22.45	220
27	22.16	220	297	14.6	180
28	24.49	220	299	25.48	180/220
31	16.63	180	300	26.45	180/220
32	21.2	180/220	303	33	220
34	26.86	180/220	304	25.54	220
36	24.31	180/220	309	22.86	220
37	34.07	220	327	24.33	220
38	26.96	220	328	31.96	220
39	33.02	220	330	25	220
41	18.46	180	333	21.69	180/220
42	33.24	220	334	20.49	180
43	25.62	180	336	42.41	220
46	27.45	220	339	29.33	220
48	24.06	180	340	22.84	220
51	29.32	220	342	26.2	220
52	27.17	220	343	23.43	220
53	25.97	220	345	26.84	220
56	49.59	220	352	21.03	180
57	44.44	220	354	9.2	180
61	13.71	180/220	360	26.74	220

62	19.6	220	374	20.57	180
63	25.98	180/220	401	25.48	220
64	30.01	220	402	27.1	220
67	28.91	220	403	25.71	220
68	36.5	220	404	25.33	220
69	27.76	220	413	24.71	180/220
72	21.06	180	414	39.43	180/220
73	16.84	180	428	20.86	180/220
74	5.18	180	429	18.56	180
77	40.11	220	430	19.28	180
78	31.03	180	441	32.06	220
81	32.62	220	442	28.44	180/220
82	23.62	220	444	38.11	220
83	31.07	180/220	457	21.44	180/220
84	19.82	180/220	459	26.49	180/220
85	24.96	220	460	33.47	180/220
86	36.97	220	500	22.5	220
87	46.57	220	507	32.66	220
88	33.64	220	511	26.3	220
89	33.8	220	515	33.8	180/220
94	28.4	180/220	518	27.24	180/220
96	24.27	220	520	26.72	180/220
97	23.62	220	556	14.32	180
100	22.14	220	558	18.55	180/220
106	5.43	180	580	11.48	180/220
107	31	220	587	22.6	220
108	30.51	180/220	589	27.12	220
109	32.88	180/220	590	25.42	220
110	29.41	180/220	599	38.58	220
111	28.41	220	600	40.22	220
112	21	220	602	24.59	180/220
114	44.67	220	604	29.9	220
121	26.24	220	608	16.93	180
125	25.87	180/220	631	17.71	180/220
128	18.59	180	633	15.42	180/220
129	19.09	180	634	15.07	180/220
131	14.21	180	636	16.07	180
133	10.69	180	637	16.44	180/220
134	20.79	180	646	41.25	220

136	30.54	220	647	44.2	220
137	21.95	220	648	41.51	220
138	30.81	220	649	40.06	180/220
147	42.1	220	650	37.79	180/220
148	55.09	220	656	16.2	180/220
156	25.07	220	659	23.09	220
159	31.81	220	677	26.87	220
161	18.8	180	678	19.95	180/220
162	34.8	180/220	696	30.94	180/220
163	45.88	220	699	31.58	180/220
181	16.9	180	702	20.67	220
182	21.22	180	705	19.09	180/220
183	19.14	180	707	21.12	180
186	18.96	180	709	25.47	180/220
187	16.51	180	711	25.13	180/220
188	21.84	180	713	13.22	180/220
189	20.8	180	714	16.39	180
197	27.84	180/220	715	17.75	180/220
201	32.65	220	736	28.2	180/220
202	28.22	220	737	30.39	220
203	19.85	180	738	27.87	180/220
204	14.15	180	739	30.75	220

Supplementary Table S5: Seed phytate content in 52 chickpea germplasm accessions**.**

Accessions	100 SDW (gm)	Phytate (mg/gm)	Cultivar Type	NCPGR90 allele
**BGD1103**	21.3	22.4	Desi	220
**BGD1105**	30.9	25.3	Kabuli	220
**BGD112**	14.7	18.7	Desi	180
**BGD72**	48.12	23.1	Desi	220
**IC296131**	8.97	12.9	Desi	180
**IC296133**	27	22.4	Desi	220
**IC449069**	30.2	23.6	Kabuli	180
**ICC11284**	14.15	21.7	Desi	220
**ICC11584**	14.52	13.6	Desi	180
**ICC1161**	13.1	14.3	Desi	220
**ICC1164**	14.5	16.8	Desi	180
**ICC12299**	31.03	22.3	Desi	220
**ICC12968**	28.5	20.3	Kabuli	220
**ICC13523**	35.78	24.9	Kabuli	220
**ICC14216**	71.71	27.9	Kabuli	220
**ICC15061**	24.95	25.4	Desi	220
**ICC15888**	14.7	14.04	Desi	180
**ICC16814**	22.74	21.4	Kabuli	220
**ICC2990**	18.5	15.03	Desi	220
**ICC4872**	21.31	20.7	Desi	220
**ICC4951**	14.9	19.6	Desi	180
**ICC4958**	25.3	19.7	Desi	180
**ICC5434**	14.6	15.7	Desi	180
**ICC5590**	15.09	12.26	Desi	220
**ICC637**	15.87	14.6	Desi	180
**ICC7323**	19.2	20.4	Desi	220
**ICC8261**	47.28	26.6	Kabuli	220
**ICC867**	17.4	17.6	Desi	180
**ICC8933**	15.53	20.8	Desi	180
**ICC9737**	19.61	17.4	Desi	220
**ICC9895**	15.5	15.53	Desi	180
**ICCL87207**	27.7	20.6	Desi	220
**ICCV1**	15.6	16.4	Desi	180
**ICCV10**	5.17	14.4	Desi	180
**ICCV88202**	23.7	18.3	Desi	180

**ICCV91106**	23.8	20.9	Desi	220
**ICCV92311**	35.80	23.1	Kabuli	220
**ICCV92337**	36.4	25.6	kabuli	220
**ICCV92944**	30.89	23.6	Desi	220
**ICCV93952**	24.1	22.6	Desi	220
**ICCV93954**	52.51	26.2	Desi	220
**ICCV93958**	24.5	21.5	Desi	220
**ICCV94954**	22.7	27.6	Desi	180
**ICCV95311**	38.2	27.5	Kabuli	220
**ICCV95334**	51.29	24.5	Kabuli	220
**ICCV96329**	44.90	26.2	Kabuli	220
**ICCX-810800**	11.62	17.8	Desi	180
**PUSA1088**	35.1	25.7	Kabuli	220
**PUSA2024**	32	23.3	Kabuli	220
**PUSA5023**	32	23.2	Kabuli	220
**PUSA5028**	39.80	23. 4	Desi	220
**PUSA547**	26.3	25.2	Desi	220

Supplementary Table S6: Seed Phytate content in F5 mapping population of (ICCV95334 × ICCX-810800).

Line No	100 SDW (gm)	Phytate (mg/gm)	Line No	100 SDW (gm)	Phytate (mg/gm)
1	29.26	26.95	161	18.8	18.51
2	31.44	27.51	163	45.88	30.85
6	30.35	24.18	166	22.65	24.53
8	18.48	16.87	171	23.97	24.04
11	34.65	24.6	176	36.76	27.16
16	32.59	25.1	181	16.9	15.03
18	26.37	22.12	182	21.22	27.8
21	23.04	19.36	184	24.19	21.98
26	26.22	26.66	186	18.96	19.64
31	16.63	18.04	191	39.22	28.01
32	21.2	23.4	196	29.11	26.17
34	26.86	26.45	197	27.84	30.28
36	24.31	21.62	199	28.43	26.31
37	34.07	28.58	213	23.71	22.12
39	33.02	29.43	239	26.07	29.43

41	18.46	21.63	258	22.12	24.82
42	33.24	30.35	270	28.93	26.87
46	27.45	23.04	299	25.48	26.45
51	29.32	24.23	304	25.54	28.51
52	27.17	27.87	308	22.86	28.72
56	49.59	29.92	328	31.96	28.43
57	44.44	30.49	334	20.49	24.68
61	13.71	14.53	339	29.33	28.01
64	30.01	21.91	345	26.84	28.01
66	23.53	21.7	360	26.74	25.53
72	21.06	21.48	371	28.58	26.87
76	27.71	22.55	396	26.46	27.73
81	32.62	24.14	401	25.48	28.29
83	31.07	26.45	403	25.71	27.94
86	36.97	24.11	414	39.43	30.78
87	46.57	30.14	429	18.56	20.63
89	33.8	22.83	441	32.06	23.12
91	31.56	24.75	443	37.19	25.53
96	24.27	19.78	511	26.3	25.74
97	23.62	23.26	515	33.8	29.92
111	28.41	24.39	516	25.71	28.86
116	16.43	21.2	520	26.72	28.15
121	26.24	27.3	557	21.29	26.87
126	23.12	26.02	558	18.55	19.21
128	18.59	20.85	580	11.48	14.82
129	19.09	20.28	588	27.12	26.52
136	30.54	27.8	599	38.58	28.36
138	30.81	27.58	600	40.22	29.92
141	24.56	21.77	631	17.71	19.5
147	42.1	25.6	648	41.51	29.64
148	55.09	30.99	658	23.09	24.04
151	43.18	29.07	680	16.38	17.65
156	25.07	23.54	697	25.34	26.17

Text S1: Primers used in this study

Primer No.	Primer sequence (5’-3’)	Purpose
Ca14825.1F	TATAGAGAGAGAAAGAGAGAGG	NCPGR90 primers
Ca14825.1R	CTAAGAGCACATACGGTTTTGT	
CaIMP9F	AGCGTGTAGCTGCTTCAAACC	CaIMP qRT-PCR
CaIMP10R	GTTTGGCGCAGAGCATCA	
GUS F	CGGGGGACTCTTGACCA	GUS qRT-PCR
GUS R	CCACAGGCCGTCGAGTT	
GFP F	CCTCGGCCACAAGTTGGAAT	GFP qRT-PCR
GFP R	CCGTTCTTTTGCTTGTCGGC	
EF 1αF	TCCACCACTTGGTCGTTTTG	qRT-PCR CaEF 1α
EF 1αR	CTTAATGACACCGACAGCAACAG	
CaIMP pro F	CCCAAGCTTTTGAGGCCCATCCATGTATGT	CaIMP promoter (1.5 Kb) cloning
CaIMP pro R	CGGGATCCTTTCATGTGGAATTGTAGAGAGAG	
